# Autoscopy of the Larynx and the Trachea

**Published:** 1897-12

**Authors:** 


**Autoscopy of the Larynx and the Trachea.** By ALFRED KIRSTEIN, M.D. Authorized Translation, by MAX THORNER, M.D. Pp. xi., 68. Philadelphia: The F. A. Davis Co. 1897.

Autoscopy of the larynx is not, as the expression implies, a process by which one can get a view of one's own larynx, but is an entirely novel method of obtaining a direct view of somebody else's larynx. The author's *brochure* on his discovery describes his instrument and the way to use it in clear and moderate language. He claims only that it is a very useful and simple procedure in certain cases. The successful employment of autoscopy for diagnosis and treatment by several observers has already afforded ample evidence of its practical utility. In a short time we believe that anyone who does not know what autoscopy is will be behind the times. We therefore cordially welcome this small but interesting work, which is so well translated by Prof. Max Thorner.

